# Predicting happiness levels of European immigrants and natives: An application of Artificial Neural Network and Ordinal Logistic Regression

**DOI:** 10.3389/fpsyg.2022.1012796

**Published:** 2022-10-31

**Authors:** Shaoming Chen, Minghui Yang, Yuheng Lin

**Affiliations:** ^1^International Business School, Guangzhou City University of Technology, Guangzhou, China; ^2^Department of Mathematics, Hong Kong Baptist University, Kowloon, Hong Kong SAR, China

**Keywords:** happiness levels, European, classification predictive modeling, immigrant, native

## Abstract

The main purpose of this paper is to investigate the happiness factors and assess the performance of machine learning techniques on predicting the happiness levels of European immigrants and natives. Two types of machine learning methods, Ordinal Logistic Regression (OLR) and Artificial Neural Network (ANN), are employed for analytical modeling. Our results with a total sample size of 196,724 respondents from nine rounds of the European Social Survey (ESS) indicate that the determinants of happiness for immigrants and natives are significantly inconsistent. Therefore, variables should be specifically selected to predict the happiness levels of these two different groups. The sensitivity analysis shows that satisfaction with life, subjective general health, and the highest level of education are the three most prominent determinants that contribute to the happiness of immigrants and natives. The overall accuracies of OLR and ANN baseline models are >80%. This can be further improved by building models for each individual country. The application of OLR and ANN implies that machine learning algorithms can be a useful tool for predicting happiness levels. The greater knowledge of migration and happiness will allow us to better understand the decision-making processes and construct more effective policies.

## Introduction

People have long been eager to find the key to open the door to happiness. For most people, happiness is the main, if not the only, goal of life. The term “happiness” is frequently used interchangeably with the scientific term “Subjective well-being” (SWB), which was first introduced by Diener (1984) and has been defined as the cognitive and affective self-assessment of an individual’s life (Diener et al., 2002). As researchers seek to understand the nature of subjective well-being, there are more and more studies on SWB and a wealth of meaningful academic achievements.

**Figure 1:**
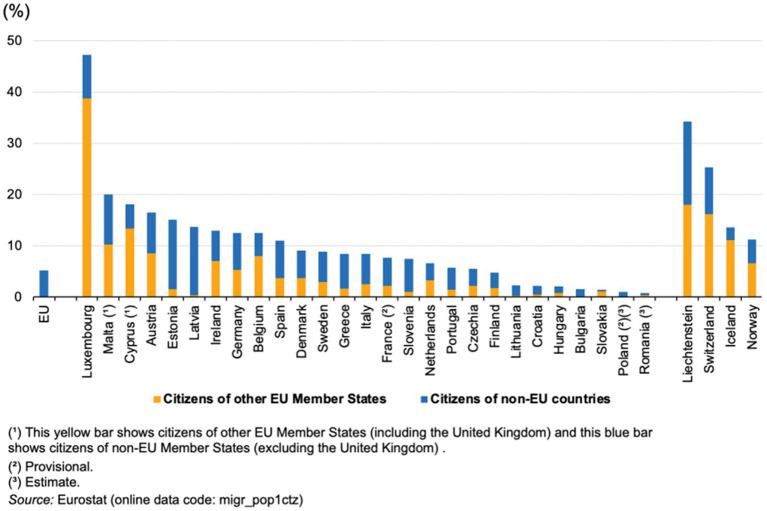
Percentage of immigrant composition in EU countries.

The number of immigrants in the European Union (EU) who are driven by a desire for a happier life is not negligible (Hendriks, 2015). According to Eurostat data, 23 million people migrated from non-EU countries to EU countries in 2020, accounting for 5.1% of the EU population (Eurostat, 2020). The proportion of immigrants from the EU Member States or non-EU countries is shown in Figure 1.

Caring for human life and well-being is the first and only legitimate goal of good governance, and a growing number of policymakers around the world are moving toward this goal. In 2019, the EU Council of Ministers asked all its member states to put people and their well-being at the center of policy design.^1^ Policymakers would benefit from understanding whether outcomes are consistent with their goals and expectations, while it can be challenging for them to forecast the happiness levels of immigrants and natives.

Most of the existing research on the question is hampered by the absence of panel data on migrants, and the approach of these studies is also limited to simple linear regression models. Hence, their findings are likely to be vulnerable to selection bias and unobserved heterogeneity. To address this gap, this article explores the factors of happiness and the accuracy of classification predictive modeling *via* analysis of data from the European Social Survey (ESS) by using the Ordinal Logistic Regression (OLR) model and the Artificial Neural Network (ANN) model. The OLR method is the most appropriate and practical technique to analyze the effect of independent variables on a rank order dependent variable, and the ANN as an alternative approach to linear regression has gained popularity in different fields (Larasati et al., 2011). Some researchers have pointed out the advantages of a supervised learning ANN against either linear or logistic regression (Dreiseitl and Ohno-Machado, 2002; Grnholdt and Martensen, 2005).

This study contributes to the literature in several ways. First, this paper enriches the understanding of happiness factors by taking more variables into consideration, which can also help to boost prediction accuracy. Second, our study extends the extant literature by providing a new perspective on happiness research *via* applying machine learning algorithms to social survey data. Third, this article sheds light on how to optimize the prediction accuracy of people’s happiness for the future studies.

The remainder of this paper is organized as follows. Section “Literature review” explains recent studies in literature parallel to the focus of the current study. Section “Methodology and data” introduces methodology and data processing, and Section “Results” reports the results and evaluation of the two algorithms. Finally, conclusions and limitations are provided in Section “Discussion”.

## Literature review

### The factors of happiness

The research about happiness or subjective well-being has been performed in different academic disciplines such as economics, psychology, and sociology. As the EAF (Ecological Acculturation Framework) suggests, immigrants’ successful adaptation to a new country is a function of the fit between the characteristics of individuals and the requirements of the settings in which they function (Salo and Birman, 2015). According to Lu and Wang (2010), the research on happiness can be summarized as the following happiness function: H=f(D,E,S,⋯) , where *H* is the dependent variable, $f\left(\cdot \right)$ is the determining equation of happiness. The independent variables are mainly composed of *D*, which are the demographic factors (e.g., age, gender, health, levels of education, religious beliefs, etc.). *E* represents the economic factors (e.g., income, unemployment, inflation, government expenditure, urbanization, etc.). *S* represents the social institution factors (e.g., democratic rights, the level of local autonomy, etc.).

The relationship between income and happiness is an eternal topic in the economics of happiness. The pioneering research of Easterlin (1974) on income and happiness arouse economists’ interest in happiness research. The now-familiar finding is that people with higher incomes are typically happier than people with less, which has been confirmed in studies of developed countries and developing countries (Easterlin and Sawangfa, 2010). In addition, cross-national studies have shown that residents of rich nations are happier than that of poor nations (Sacks et al., 2012).

Unemployment has a significant negative impact on happiness (Štreimikienė and Grundey, 2009; Krause, 2014). First, unemployment can lead to a decrease in income, thereby reducing the quality of life. Second, unemployment can cause psychological stress. Unemployed people have negative feelings of depression, anxiety, and even shame, leading to a loss of self-esteem and social isolation. The analysis of the national unemployment rate shows that when the unemployment rate of the country is low, people tend to be happier (Stanca, 2010).

Some studies on the happiness of people living in different areas find that living in a large city has a negative effect on happiness. On the contrary, living in rural or suburban regions has a positive effect (Lenzi and Perucca, 2020). The main explanations for this conclusion are: (1) people in cities are full of uncertainty about the future, and fear of unemployment; (2) The cost of living in urban areas is higher; (3) social and environmental issues, such as crime rates, traffic congestion, and population density.

Self-assessed health, have a strong positive correlation with SWB (MacKerron, 2012). The relationship between health status and SWB is bidirectional, with physical health being a determinant of happiness. Older people with illnesses such as coronary heart disease, arthritis, and chronic lung disease show an increase in depression, a decrease in hedonism, and an impaired sense of well-being; psychological well-being also has a protective effect on health, reducing some psychological illnesses to a certain extent and leading to a longer life (Steptoe et al., 2015).

MacKerron (2012) found that the levels of positive emotions and happiness of women are higher than that of men. The reasons for this difference are mainly: (1) Women’s income levels have increased significantly and income is an important factor in happiness; (2) Discrimination against women’s work, abilities are decreasing and social recognition has a positive impact on enhancing SWB; (3) Women are better at controlling the negative impact of emotions than men (Chong and Yue, 2020); (4) The increasing educational attainment of women (Triandafyllidou and Isaakyan, 2016) and (5) The improved social-labor situation (Elgorriaga et al., 2020). It is worth noting that women aged above 50 tend to have a lower level of happiness because of the transition to menopause (Beutel et al., 2009). Accumulating resources in personal (e.g., self-esteem) and social (e.g., income, employment, and partnership) and away from anxiety and depression play crucial roles in maintaining the happiness of aging women (Smith-DiJulio et al., 2008; Beutel et al., 2009).

There is a “U-shaped” relationship between age and SWB, meaning that happiness decreases and then increases as age increases (Steptoe et al., 2015; Clark, 2018). The socioemotional selectivity theory explains that as people age, they accumulate emotional wisdom that makes them choose to retain more emotionally satisfying events, friendships, and experiences. A moderate increase in positive emotional experiences may offset the increase in physical pain. Thus, despite the fact that older people face reduced income, lower social status, and increased mortality, their happiness does not necessarily decline (Steptoe et al., 2015).

### The effect of migration on happiness

The motivation for migration is to pursue a better life, while the outcomes of the choice are difficult to predict. Researchers from various disciplines have long been interested in the role that migration play in people’s happiness. There are two main questions that are being investigated: (1) Do immigrants become happier after migration? and (2) Are immigrants as happy as natives?

A number of studies show that immigrants are likely to be happier after migration (Cuellar et al., 2004; Erlinghagen, 2012), but the specific migration flows can make a difference. According to the report of the International Organization for Migration (IOM) (2013), migration has a negative effect on the immigrants’ happiness when they flow to developing countries. In addition, Western Europeans who move to undeveloped areas such as Southern Europe suffer from a decline in the happiness level (Bartram, 2015). In contrast, Eastern Europeans tend to be happier when they migrate to Western Europe (Bartram, 2013).

From the aspect of migration timing, Guedes Auditor and Erlinghagen (2021) found some evidence for an increase in individual SWB in the course of migration and a significant decrease in SWB 1–2 years before migration (Erlinghagen, 2016). Lower life satisfaction and higher perceived isolation reported from those couples which men immigrate in advance of their female partners (Erlinghagen, 2021). However, this trailing-wife-hypothesis on international migration couples not only relies on small immigrant samples (KIing-O’Riain and Chiyoko, 2015) and specific immigrant group (e.g., high-skilled immigrants; Cangia et al., 2018), but also is restricted by specific migration flows (Kõu et al., 2015; Mayes and Koshy, 2018).

Concurrently, a few studies show that immigrants occasionally reach the happiness levels of natives (Cuellar et al., 2004; Rasmi et al., 2012). The possible causes of this phenomenon are that (1) the sample sizes of immigrants are too limited to detect significant differences (Hendriks, 2015) and (2) the included time-variant controls (e.g., income and health) offset the potential gaps (Obućina, 2013). Whether migration decisions bring happiness or not depends on several aspects, and typically, immigrants might not reach the same happiness levels as natives. To address this situation, scholars and policymakers need to help immigrants make optimal decisions to develop a society that incorporates thriving immigrants.

When formulating policies related to improving public happiness, it can be more precise with a prediction system. The government will be able to predict the happiness levels of people by inputting their characteristics into the prediction system. This makes policies vary from person to person, thereby saving public resources and improving efficiency. For both immigrants and natives to benefit from the policy and improve their happiness, machine learning algorithms can be employed to predict their happiness levels.

In our study, we establish the OLR model and the ANN model *via* using the ESS data to assess the performance of machine learning techniques on predicting the happiness levels of European immigrants and natives. Furthermore, the differences in happiness factors between these two groups are investigated through single-country and cross-country modeling, and the degree of the relative importance of happiness factors is assessed with sensitivity analysis.

## Methodology and data

### Methodology

Most of the machine learning algorithms applied in happiness research use unstructured data in computer science, such as predicting the happiness levels through facial features in pictures (Li et al., 2016) and the textual data on Twitter (Islam and Goldwasser, 2020). Some studies used machine learning techniques on survey data. A cross-sectional survey in Spain with 823 samples shows that the performance of Deep Neural Network (DNN) in predicting the level of happiness is better than that of the traditional method (Pérez-Benito et al., 2019). In addition, a study using two rounds of ESS data to investigate the German national vote also proved that neural network is superior to other methods (Weber et al., 2018).

In this study, the response variable *Y* is ordinal, representing three levels of happiness (unhappy, neutral, happy). Since the ordinality of the response variable, we use OLR for the first analysis. This is one of the variations of logistic regression used for predicting an ordinal response variable *Y*. The advantage of OLR is that it does not assume a spacing between levels of *Y*. In other words, even if the levels of *Y* are recorded as 1, 5, 10, it can have the same regression coefficients and *p*-values from a response variable at levels of 0, 1, 2. Therefore, only the values of rank-ordering of *Y* are used in the ordinal model.

The most commonly used OLR model is called the proportional odds (PO) model. The PO model for a response variable *Y* with levels 0,1,⋯,k is stated as follows:


(1) $$Prob\left[P\left(Y\geq j|X\right)\right]=\frac{1}{1+\exp{\left[-\left(\alpha_{j}+\beta X\right)\right]}^{\prime }}$$

where j=1,2,⋯,k ; α is the intercepts with the number of k , and β is the regression coefficients. For fixed j , the model is an ordinary logistic model for the event Y≥j . Using a common vector of β to correlate the probability for varying j , the PO model can perform parsimonious modeling of the distribution of Y (Harrell, 2015).

The ANN model is one of the most popular machine learning algorithms applied in social science studies. Furthermore, the ANN model is also an isomorph with binary classification logistic regression when it has zero hidden nodes (also known as “neurons”; Boulle et al., 2001). Therefore, it is possible to compare the performance of the ANN model with the OLR model. Some studies pointed out that the ANN model appeared to be more powerful in predicting the level of happiness versus the LR models (Dreiseitl and Ohno-Machado, 2002; Grnholdt and Martensen, 2005).

**Figure 2:**
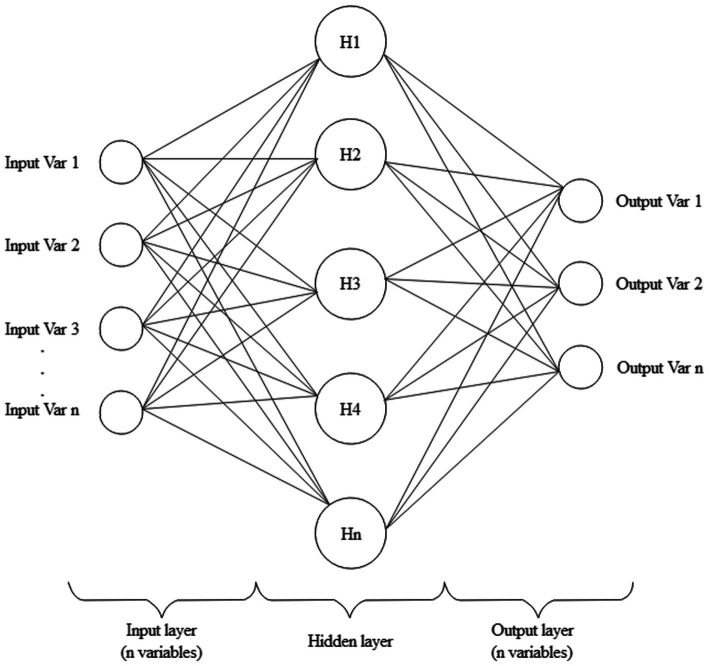
The “vanilla” neural network.

The simplest and the most widely used structure in the ANN model consists of three layers: input, hidden and output layers, as shown in Figure 2. When the ANN model has a single hidden layer, this structure is also known as a “vanilla” neural network.

The first layer includes inputs that represent the feature (independent) variables, and the last layer includes outputs that are the response (dependent) variables. The number of nodes in the output layer is equal to the number of the classification categories of the model. The nodes of the hidden layer connect the input and output layers, where the network takes in weighted inputs and produces the outputs through an activation function. When the ANN contains one or more hidden layers, it is a multilayer perceptron.

The ANN model is a combination of function composition and matrix multiplication. The expression is


(2) $$g\left(x\right)=f^{L}\left(W^{L}f^{L-1}\left(W^{L}\cdots f^{1}\left(W^{1}x\right)\cdots \right)\right)$$

where x is the input. *L* is the number of layers. $f^{L}$ is the activation function at layer l . $W^{L}=\left(w_{jk}^{l}\right)$ are the weights between *k*-th node in layer l−1 and *j*-th node in a layer.

### Data processing and descriptive statistic

The data for this study are from the European Social Survey (ESS, 2020). This is a cross-national survey conducted every 2 years since its establishment in 2001. To achieve a large sample size, the data in this study is pooled across all ESS from round 1 in 2001 to the latest round 9 in 2018. For the cross-national comparison, we select 15 countries into account because the data of other countries are absent in at least one round of the survey. After the selection of the ESS database, the total sample size is 255,824 from nine rounds of surveys in 15 countries.

Since most variables in survey data are categorical variables and represent answers to survey questions. Some samples should be identified as invalid by values as 6/66 (i.e., not applicable), 7/77 (i.e., refusal), 8/88 (i.e., do not know), and 9/99 (i.e., no answer). The immigrants and natives are identified by the answer to the question that “Are you born in this country?” (Bartram, 2013). After defining and deleting these invalid values, the dataset remains 76.8982% of the original case. There are 196,724 samples in total, of which 179,324 are natives and 17,400 are immigrants.

The response variable of this study is happiness. The question C1 “How happy are you” in all survey rounds has answers ranging from 0 to 10. The measurement of happiness is a hot topic with many methods that contain multi-item scales, such as the Subjective Fluctuating Happiness Scale (SFHS) and the Subjective Authentic–Durable Happiness Scale (SA–DHS) have gained popularity, with which some studies provide insights into the general construct of happiness (Dambrun et al., 2012; Monacis et al., 2021). For our study that includes the analysis of different countries, using a single measure can minimize the potential for various interpretations of specific dimensions. Thus, we use a single-item scale for comparisons across different countries and cultures (Abdel-Khalek, 2006; Graham, 2012). It is meaningless to use 11 answer values to classify the happiness levels directly. Therefore, this study defines happiness into three levels: Unhappy (values ranging from 0 to 3), Neutral (values ranging from 4 to 6), and Happy (values ranging from 7 to 10).

The feature variables are selected by the forward stepwise regression method, where the baseline models are ordinal logistic regression (OLR) models of total native group and total immigrant group. Finally, 13 selected feature variables in the models below represent the determinants that are significantly associated with happiness (Bartram, 2012).

The mean and standard deviation of variables are listed in Table 1, which shows that immigrants are happier than natives. On top of that, immigrants have more faith in the politics and economy of countries. However, the unemployment rate of immigrants is higher than the natives, and their income is lower than natives.

**Table 1:** Variable definition and descriptive statistics.

Variables	Variable definition	Variable assignment	Total	Native	Immigrant
happy	The level of happiness	10-point scale from extremely unhappy to extremely happy	7.4732 (0.0058)	7.4702 (0.0060)	7.5032 (0.0209)
agea	Age of respondent	Respondents’ age in each survey round	47.9667 (0.0563)	48.2520 (0.0592)	45.1118 (0.1784)
trstprl	Trust in the country’s parliament	10-point scale from no trust at all to complete trust	4.4768 (0.0078)	4.4085 (0.0081)	5.1602 (0.0280)
stflife	Satisfaction with life	10-point scale from extremely dissatisfied to extremely satisfied	7.0694 (0.0071)	7.0741 (0.0074)	7.0222 (0.0254)
stfeco	Satisfaction with the country’s economic situation	10-point scale from extremely dissatisfied to extremely satisfied	4.5101 (0.0078)	4.4532 (0.0080)	5.0793 (0.0279)
domicil	Domicile, respondent’s description	1 for a big city; 2 for suburbs or outskirts of the big city; 3 for town or small city; 4 for the country village; 5 for farm or home in countryside	2.9505 (0.0036)	2.9896 (0.0037)	2.5585 (0.0124)
sclmeet	Frequency of socially meet with friends, relatives, or colleagues	1 for never; 2 for less than once a month; 3 for once a month; 4 for several times a month; 5 for once a week; 6 for several times a week; 7 for every day	4.9397 (0.0048)	4.9463 (0.0050)	4.8733 (0.0172)
health	Subjective general health	5-point scale from very good to very bad	2.2235 (0.0029)	2.2317 (0.0030)	2.1417 (0.0099)
rlgdgr	Religious piety	10-point scale from “not at all religious” to “very religious”	4.4223 (0.0099)	4.3252 (0.0102)	5.3940 (0.0356)
ppltrst	Most people can be trusted, or you cannot trust anyone	10-point scale from “you cannot trust anyone” to “most people can be trusted”	5.0249 (0.0072)	5.0178 (0.0075)	5.0954 (0.0249)
income	Weekly total net income of household, all sources	J Less than £208 (1st decile) R £208 to under £279 (2nd decile) C £279 to under £348 (3rd decile) M £348 to under £416 (4th decile) F £416 to under £499 (5th decile) S £499 to under £590 (6th decile) K £590 to under £704 (7th decile) P £704 to under £858 (8th decile) D £858 to under £1,124 (9th decile) H £1,124 or more (10th decile)	5.8403 (0.0088)	5.8772 (0.0091)	5.4715 (0.0312)
eisced	Highest level of education, ES – ISCED	Not possible to harmonise into ES-ISCED ES-ISCED I, less than lower secondary ES-ISCED II, lower secondary ES-ISCED IIIb, lower tier upper secondary ES-ISCED IIIa, upper tier upper secondary ES-ISCED IV, advanced vocational, sub-degree ES-ISCED V1, lower tertiary education, BA level ES-ISCED V2, higher tertiary education, > = MA level	3.4252 (0.0116)	3.3886 (0.0116)	3.7919 (0.0529)
gndr	Gender	1 for male, 2 for female	1.5026 (0.0016)	1.5023 (0.0017)	1.5053 (0.0057)
unemploy	Main activity, last 7 days, coded as unemployed	0 for employed, 1 for unemployed	0.0546 (0.0007)	0.0516 (0.0008)	0.0842 (0.0032)

Last three columns present the mean of each variable in the total sample group, native sample group and immigrant sample group; The standard deviation is in parentheses.

## Results

### Ordinal logistic regression

On the basis of the data pre-processing, the feature variables are determined. Two OLR models with the total samples of native group and immigrant group are selected as the baseline models of this study. The data in each model is divided into a training dataset (70%) and a test dataset (30%) to train the models. Then, the performance of trained models is tested.

A more comprehensive setting of OLR models should be considered. The OLR models are only valid when the assumptions are satisfied. The Variance Inflation Factor (VIF) tests should be performed to confirm there is no multi-collinearity. Since all the VIF for each independent variable in the OLR models is <10, there are no multi-collinearity problems. The mean values of VIF for each model are shown in Tables 2, 3. Moreover, the Brant test should be conducted to test the proportional odds assumption. We conclude that the assumption holds since the probability for all variables in the models is >0.05. Therefore, the proportional odds assumption is not violated, and the models are valid.

**Table 2:** The comparison of happiness determinants across nations in native group.

Variables	Total	BE	FI	FR	DE	HU	IE	NL	NO	PL	PT	SI	ES	SE	CH	GB
essround	0.0378^***^ (11.7923)	0.0546^***^ (3.3494)	0.0324 (1.5326)	0.0233^*^ (1.8351)	0.0780^***^ (6.3021)	−0.0296^***^ (−2.6403)	−0.0062 (−0.3564)	0.0560^***^ (2.8931)	0.0607^***^ (3.1993)	0.0197 (1.5583)	0.0685^***^ (4.5621)	0.0556^***^ (3.7796)	0.0458^***^ (2.9173)	0.0215 (1.0429)	0.0076 (0.2964)	0.0380^***^ (2.6384)
trstprl	0.0347^***^ (9.1864)	0.0170 (0.8461)	0.0027 (0.1311)	0.0261^*^ (1.8784)	0.0419^***^ (3.2568)	0.0404^***^ (3.2653)	0.0355^**^ (2.3061)	0.0335 (1.2568)	0.0082 (0.3917)	0.0045 (0.3149)	−0.0057 (−0.3826)	0.0565^***^ (3.1541)	0.0234 (1.4291)	0.0191 (0.9551)	0.0381 (1.3760)	0.0271^*^ (1.7372)
stflife	0.6598^***^ (146.8335)	0.8618^***^ (33.1798)	0.8791^***^ (33.2819)	0.5465^***^ (36.4364)	0.6870^***^ (44.7048)	0.5180^***^ (35.0615)	0.6938^***^ (37.7012)	1.0270^***^ (29.8359)	0.9633^***^ (35.0172)	0.6245^***^ (37.8911)	0.5016^***^ (27.9371)	0.6597^***^ (31.0726)	0.7300^***^ (33.7687)	0.9915^***^ (34.1622)	0.7869^***^ (24.9163)	0.7847^***^ (40.8465)
stfeco	0.0080^*^ (1.9503)	0.0379 (1.6426)	0.0408^*^ (1.7799)	−0.0242 (−1.4259)	−0.0120 (−0.8472)	0.0110 (0.7211)	−0.0153 (−0.9975)	−0.0039 (−0.1302)	−0.0154 (−0.7325)	0.0934^***^ (5.6375)	0.0336^*^ (1.7318)	0.0529^***^ (2.7119)	−0.0373^**^ (−1.9877)	0.0352 (1.6003)	0.0605^**^ (2.0595)	0.0453^***^ (2.6453)
domicil	0.0298^***^ (4.2471)	0.0656^*^ (1.8190)	0.0214 (0.7017)	0.0016 (0.0653)	0.0375 (1.5537)	−0.0556^**^ (−2.3610)	0.0883^***^ (3.5837)	0.0748^*^ (1.9332)	0.0069 (0.2174)	−0.0411 (−1.5475)	−0.0309 (−1.0803)	−0.0213 (−0.6294)	0.0066 (0.2151)	0.0267 (0.7592)	0.1058^**^ (2.1047)	0.0090 (0.2536)
eisced	0.0010 (0.3912)	0.0116 (0.8315)	0.0165 (0.9346)	−0.0220^*^ (−1.7976)	0.0194 (1.3651)	0.0403^**^ (2.2379)	0.0248 (1.4598)	−0.0028 (−0.1624)	−0.0242^*^ (−1.6812)	0.0640^***^ (3.7511)	−0.0210 (−1.4063)	0.1166^***^ (3.5623)	0.0164 (0.7970)	−0.0113 (−0.7176)	0.0517^*^ (1.8719)	−0.0011 (−0.1833)
sclmeet	0.1302^***^ (26.0464)	0.0955^***^ (3.6490)	0.1188^***^ (4.2148)	0.1333^***^ (6.8004)	0.0685^***^ (3.6120)	0.0961^***^ (5.5390)	0.1317^***^ (6.6011)	0.1533^***^ (4.5119)	0.1364^***^ (4.3089)	0.0784^***^ (3.9246)	0.1147^***^ (5.7682)	0.1047^***^ (4.2741)	0.1165^***^ (5.2052)	0.0485 (1.5853)	0.1485^***^ (3.7683)	0.1205^***^ (6.0669)
health	−0.3671^***^ (−40.5116)	−0.3176 (−6.3157)	−0.3903^***^ (−7.1059)	−0.3522^***^ (−10.1494)	−0.3259^***^ (−10.1404)	−0.4556^***^ (−12.8017)	−0.3140^***^ (−7.7991)	−0.3789^***^ (−6.2062)	−0.2518^***^ (−5.3415)	−0.3948^***^ (−10.0312)	−0.4277^***^ (−9.9703)	−0.3378^***^ (−7.1357)	−0.3304^***^ (−7.7378)	−0.3870^***^ (−7.7137)	−0.2801^***^ (−4.2160)	−0.3721^***^ (−10.6020)
rlgdgr	0.0205^***^ (7.5822)	−0.0088 (−0.6457)	0.0535^***^ (3.3517)	0.0148 (1.5503)	0.0262^***^ (2.7962)	0.0190^**^ (1.9967)	0.0545^***^ (4.0521)	0.0204 (1.2770)	0.0375^**^ (2.2655)	0.0778^***^ (6.1028)	0.0887^***^ (6.4669)	0.0248^*^ (1.8647)	0.0270^**^ (1.9726)	0.0543^***^ (3.3525)	0.0205 (1.0950)	0.0171 (1.4144)
gndr	0.0510^***^ (3.2055)	−0.0878 (−1.0930)	0.2062^**^ (2.4437)	0.1167^**^ (2.0804)	−0.0237 (−0.4439)	0.1411^***^ (2.6090)	−0.0164 (−0.2520)	0.0517 (0.5408)	−0.1558^*^ (−1.8624)	−0.0349 (−0.5794)	−0.1210^*^ (−1.8141)	0.0884 (1.1869)	−0.1067 (−1.4806)	0.0606 (0.7329)	0.0823 (0.7857)	0.0655 (0.9977)
agea	0.0008 (1.6235)	0.0003 (0.1189)	−0.0004 (−0.1498)	−0.0035^**^ (−1.9772)	−0.0017 (−1.0339)	−0.0046^**^ (−2.4345)	0.0062^***^ (2.8533)	0.0068^**^ (2.3012)	0.0041 (1.5496)	−0.0041^*^ (−1.9581)	−0.0124^***^ (−5.8335)	−0.0124^***^ (−5.0185)	−0.0122^***^ (−4.8809)	0.0044^*^ (1.8131)	0.0002 (0.0534)	0.0124^***^ (5.8812)
ppltrst	0.0625^***^ (16.6743)	0.0434^**^ (2.2851)	0.1095^***^ (5.0721)	0.0751^***^ (5.3491)	0.0474^***^ (3.7961)	0.0396^***^ (3.2605)	0.0817^***^ (5.8471)	0.0528^**^ (2.1427)	0.0770^***^ (3.3913)	0.0390^***^ (2.8982)	0.0080 (0.5390)	0.0592^***^ (3.6804)	0.0682^***^ (3.9400)	0.0122 (0.5817)	0.0803^***^ (3.0972)	0.0807^***^ (5.0508)
income	0.0852^***^ (26.5577)	0.0892^***^ (4.7897)	0.1076^***^ (6.1304)	0.0970^***^ (8.2055)	0.1088^***^ (9.6927)	0.0542^***^ (4.6152)	0.0858^***^ (6.5205)	0.1076^***^ (5.3698)	0.0837^***^ (5.1824)	0.0570^***^ (4.0353)	0.1259^***^ (7.5641)	0.0867^***^ (4.3472)	0.0241 (1.4402)	0.1306^***^ (7.9066)	0.0781^***^ (3.5999)	0.0477^***^ (3.8551)
unemploy	−0.0690^**^ (−2.2620)	−0.2204 (−1.4877)	−0.0269 (−0.1824)	−0.2045^*^ (−1.8663)	0.0012 (0.0116)	−0.2488^**^ (−2.0857)	−0.2960^***^ (−2.8517)	0.1471 (0.6617)	−0.0559 (−0.2688)	−0.1127 (−0.9939)	−0.2132^*^ (−1.8340)	−0.1240 (−0.7914)	−0.0466 (−0.3930)	0.0681 (0.3864)	−0.4344^*^ (−1.7385)	−0.1155 (−0.8232)
AIC	108,034.5900	4,875.7320	4,878.7240	9,246.121	10,985.6700	10,108.5700	7,845.9920	3,720.3820	4,554.5320	8,111.2280	7,249.6470	5,366.5740	5,904.6630	4,630.6900	3,064.0570	7,502.1000
VIF_avg	1.2219	1.1725	1.4162	1.2424	1.2592	1.3087	1.3325	1.1852	1.1948	1.2728	1.2678	1.2514	1.2362	1.2852	1.2009	1.1929
N	179,324	12,023	15,716	13,363	18,605	9,824	12,613	12,832	12,486	10,671	8,032	7,882	10,093	12,358	9,077	13,746

***, ** and * indicate *p*-values of coefficients are significant at the 1%, 5%, and 10% levels, respectively; The *t* statistics is in parentheses; N represents the number of samples; The top row represents names of countries, and the full names of each investigated country are shown in Table A1 in the Appendix.

**Table 3:** The comparison of happiness determinants across nations in immigrant group.

Variables	Total	BE	FI	FR	DE	HU	IE	NL	NO	PL	PT	SI	ES	SE	CH	GB
essround	0.0402^***^ (4.0307)	−0.0419 (−1.0082)	0.0809 (0.7056)	0.0811^**^ (1.9837)	0.0446 (1.2921)	0.0741 (0.9864)	−0.0685 (−1.2313)	0.0784 (1.6192)	0.1783^***^ (3.0291)	0.0453 (0.3667)	0.1800^***^ (2.9921)	0.0894^*^ (1.9413)	0.0910^*^ (1.8292)	0.0459 (1.0270)	−0.0256 (−0.6122)	0.0170 (0.4636)
trstprl	0.0516^***^ (4.7765)	0.0053 (0.1200)	−0.0653 (−0.7116)	0.0653^*^ (1.6971)	−0.0154 (−0.4334)	−0.0168 (−0.1381)	0.0402 (0.9549)	−0.0006 (−0.0103)	−0.0474 (−0.8298)	−0.0491 (−0.3854)	0.0687 (1.0988)	0.1108^**^ (2.0397)	0.0522 (1.0676)	0.0274 (0.5839)	−0.1290^***^ (−2.8731)	0.0945^**^ (2.3867)
stflife	0.5842^***^ (44.8891)	0.6100^***^ (11.1564)	0.7098^***^ (5.6861)	0.5286^***^ (12.0882)	0.6621 (15.2469)	0.4404^***^ (3.4230)	0.7650^***^ (14.6597)	0.6828^***^ (9.5583)	0.8239^***^ (10.9383)	0.7428^***^ (4.7286)	0.5227^***^ (7.1944)	0.6164^***^ (10.0707)	0.6643^***^ (11.4945)	0.6683^***^ (10.9567)	0.6003^***^ (12.4611)	0.7009^***^ (13.5915)
stfeco	0.0055 (0.4728)	0.0160 (0.3142)	0.0631 (0.5922)	−0.0047 (−0.1012)	0.1162 (2.9171)	−0.0080 (−0.0609)	0.1100^**^ (2.5316)	0.1732^***^ (2.6826)	0.0594 (0.9200)	0.1612 (1.0781)	−0.1514^*^ (−1.7711)	0.0324 (0.5763)	−0.0020 (−0.0371)	0.0334 (0.6245)	0.3229^***^ (6.5413)	0.1305^***^ (2.8757)
domicil	0.0583^***^ (2.7643)	0.0770 (0.9869)	0.1646 (1.0531)	0.0544 (0.7723)	0.1356 (1.8205)	0.2056 (0.9899)	0.0553 (0.7704)	0.1148 (1.2283)	0.1015 (1.0119)	−0.2110 (−0.7075)	−0.0026 (−0.0222)	−0.2417^**^ (−2.3553)	0.0310 (0.3531)	−0.0297 (−0.3108)	0.1301 (1.6355)	−0.1249 (−1.4044)
eisced	0.0101^*^ (1.9025)	0.0363^*^ (1.8700)	−0.0750 (−0.7380)	−0.1366^***^ (−3.0172)	0.0575 (1.8135)	0.3672 (1.6343)	0.1588^***^ (3.2170)	0.0400 (1.6082)	−0.0764 (−1.0171)	0.0194 (0.1027)	0.0085 (0.2059)	−0.1411 (−1.3153)	0.0013 (0.0335)	−0.0172 (−1.1476)	0.0160 (0.6209)	0.0328^**^ (2.1968)
sclmeet	0.1577^***^ (10.3595)	0.2218^***^ (3.6904)	0.2246^*^ (1.7704)	0.1941^***^ (3.4857)	0.0238 (0.4356)	0.1192 (0.8988)	0.2142^***^ (3.9331)	0.1795^**^ (2.3246)	0.0873 (1.0792)	0.1066 (0.6388)	0.1312 (1.5689)	0.1810^**^ (2.3082)	−0.0017 (−0.0245)	0.0909 (1.3076)	0.1382^**^ (2.2666)	0.1838^***^ (3.2729)
health	−0.3017^***^ (−11.1479)	−0.4552^***^ (−4.0943)	−0.6173^**^ (−2.5417)	−0.2656^***^ (−2.5895)	−0.3654 (−3.8594)	−0.4656 (−1.4084)	−0.4729^***^ (−4.4338)	−0.1380 (−0.9265)	−0.4645^***^ (−3.7313)	−1.3831^***^ (−3.9887)	−0.8111^***^ (−4.5718)	−0.7208^***^ (−4.9177)	−0.2934^**^ (−2.1240)	−0.3079^**^ (−2.5087)	−0.5207^***^ (−5.0350)	−0.0775 (−0.7334)
rlgdgr	0.0124 (1.5839)	0.0305 (1.0410)	−0.0465 (−0.7293)	0.0195 (0.6902)	0.0080 (0.2973)	0.0655 (0.7809)	0.0485 (1.5677)	−0.0249 (−0.6026)	0.0046 (0.1090)	0.0843 (0.8654)	0.1172^**^ (2.3779)	−0.0661 (−1.6271)	0.0457 (1.2204)	0.1469^***^ (4.2575)	−0.0327 (−1.1532)	0.0080 (0.2761)
gndr	0.0539 (1.1224)	0.1857 (0.9873)	−0.1620 (−0.4245)	−0.1536 (−0.9111)	0.2296 (1.4504)	0.6304 (1.2300)	0.3440^*^ (1.9270)	−0.0652 (−0.2802)	−0.3237 (−1.2766)	0.0952 (0.1830)	−0.1002 (−0.3772)	0.3587 (1.4882)	0.0711 (0.3253)	−0.2207 (−1.0682)	−0.0320 (−0.1893)	−0.0088 (−0.0512)
agea	0.0050^***^ (3.0625)	0.0056 (0.8833)	0.0065 (0.4422)	0.0009 (0.1642)	−0.0023 (−0.4250)	−0.0292^***^ (−2.7981)	0.0069 (0.9595)	−0.0051 (−0.5920)	−0.0139 (−1.6093)	0.0199 (0.9955)	−0.0137 (−1.3981)	−0.0078 (−0.7946)	−0.0125 (−1.4974)	−0.0064 (−0.9360)	0.0037 (0.6393)	−0.0008 (−0.1342)
ppltrst	0.0619^***^ (5.5190)	0.1021^**^ (2.2673)	0.1393 (1.3461)	0.0702^*^ (1.6917)	0.0773 (2.0917)	0.1118 (1.0322)	0.0982^**^ (2.4627)	0.1552^***^ (2.6872)	0.1205^*^ (1.8321)	0.1554 (1.3472)	0.0551 (0.9005)	0.1223^***^ (2.6205)	0.0504 (0.9990)	0.1972^***^ (4.1387)	0.0908^**^ (2.2624)	0.0292 (0.7051)
income	0.0858^***^ (8.7835)	0.0927^**^ (2.1345)	0.1771^**^ (2.2606)	0.1041^*^ (2.7730)	0.0926 (2.5668)	0.0950 (0.8791)	0.0512 (1.3475)	0.0940^*^ (1.9569)	0.1557^***^ (3.0315)	0.1714 (1.3948)	0.1181^**^ (1.9906)	0.0404 (0.5745)	0.0709 (1.4716)	0.0989^**^ (2.4099)	0.1047^***^ (2.9786)	0.0706^**^ (2.3790)
unemploy	−0.1084 (−1.4122)	−0.4762^*^ (−1.7836)	0.9935 (1.2859)	−0.6143^**^ (−2.2140)	0.4485 (1.6395)	−1.6495^*^ (−1.6607)	−0.4660^**^ (−2.0544)	0.2710 (0.5946)	−0.4107 (−0.9711)	4.1926^**^ (2.5568)	−0.7237^*^ (−1.8844)	−0.5915 (−1.3673)	−0.0498 (−0.1838)	−0.1916 (−0.4899)	−0.6687^**^ (−2.0630)	0.0764 (0.2353)
AIC	11,423.5800	923.8731	265.5296	1,053.7270	1,146.3970	163.0373	1,054.0710	619.8967	521.0202	120.9463	515.1255	560.0765	694.4052	765.8601	1,079.859	984.4060
VIF_avg	1.1784	1.1714	1.3904	1.2138	1.1780	1.3778	1.2797	1.1701	1.1963	1.4495	1.2472	1.1990	1.1575	1.2198	1.1826	1.1903
N	17,400	1,534	517	1,398	1,768	186	1,744	12,832	1,166	125	601	797	1,033	1,501	2,275	1,589

***, ** and * indicate *p*-values of coefficients are significant at the 1%, 5%, and 10% levels, respectively; The *t* statistics is in parentheses; N represents the number of samples; The top row represents names of countries, and the full names of each investigated country are shown in Table A1 in the Appendix.

When analyzing the ESS data, survey weights should be taken into account. If there are no weights, the estimation may be biased and over-fitted (Kaminska, 2020). Therefore, population weights and design weights are employed for the regressions of multiple countries. However, only design weight is employed for the regressions of a single country (Bartram, 2019). The survey package is used to specify sample design in R software and construct the ordinal logistic regression. In addition, we use the survey round as a variable in all models to mitigate the influence of time and events, because the investigated period encompasses significant events, such as the economic crisis. We use the same set of feature variables and other settings in the analysis of all models and change only the sample size from total samples to the sample of the individual country. This can validate the consistency in the determinants of happiness.

Tables 2, 3 demonstrate that there are diverged in the happiness factors between the total native and immigrant groups. *Stfeco, rlgdgr, gndr*, and *unemploy* significantly influence the happiness of the total native group. However, *agea* and *eisced* are determinants of happiness for the total immigrant group. The results of the two baseline models show a difference between immigrants and natives in determinants of happiness.

We compare the native group with the immigrant group of their total samples in the two baseline models. The comparison at the level of individual countries can be investigated with separate ordinal logistic regression. The results of separate regressions show that *stflife, sclmeet, ppltrst, income* and *health* are the determinants of happiness of the native groups in most countries. Comparing the results of total samples with that of the individual countries’ samples, the differences in the determinants of happiness in the native groups are relatively small. However, the determinants of the immigrant group fluctuate drastically between different countries. Only *stflife* and *health* are significant to happiness in immigrant groups of most countries. Therefore, the determinants of happiness are inconsistent because they vary from the total samples to the samples of individual countries. In other words, the factor may be not significant to happiness when changing the samples in the model. The values of the Akaike Information Criterion (AIC) in each OLR model show that it is better to set up the model for each individual country. In addition, the AIC values of both native and immigrant groups in the model of individual countries are less than that of the total samples in the baseline models.

One of the main purposes of our research is to predict the happiness levels of immigrants and natives. Therefore, the performance metrics of accuracy, F1-score, precision, and recall are employed to assess the models. Considering the macro metrics can treat the importance of each sample equally, we adopt the macro average of F1-score, precision, and recall to evaluate the multi-class classification. In other words, it assigns equal weight to all data points. The mltest package is employed to calculate the performance metrics of multiclass classification based on the confusion matrix. The calculation of the macro F1-score, precision and recall are as follows:


(3) $${\mathrm{Precision}}_{\mathrm{macro}}=\frac{1}{\left|Y\right|}\overset{\left|Y\right|}{\underset{i=1}{\sum }}\frac{TP_{i}}{TP_{i}+FP_{i}}$$

(4) $${\mathrm{Recall}}_{\mathrm{macro}}=\frac{1}{\left|Y\right|}\overset{\left|Y\right|}{\underset{i=1}{\sum }}\frac{TP_{i}}{TP_{i}+FN_{i}}$$

(5) $$F1_{\mathrm{macro}}=2\frac{{\mathrm{Precision}}_{\mathrm{macro}}\cdot {\mathrm{Recall}}_{\mathrm{macro}}}{{\mathrm{Precision}}_{\mathrm{macro}}+{\mathrm{Recall}}_{\mathrm{macro}}}$$

where the happiness is *Y* in 3 levels. i represents the i−th class. The confusion matrix of i−th class against other classes is used to calculate the $TP_{i}$ , $FP_{i}$ , and $FN_{i}$ . These indicate the True Positive Rate of i−th class, the False Positive Rate of i−th class, and the False Negative Rate of i−th class, respectively.

The performance assessments of the native group are shown in Table 4. For the OLR models in the native group, macro metrics of most models are above 50%. The accuracy rates of OLR models for Belgium (i.e., BE), Finland (i.e., FI), Netherlands (i.e., NL), Norway (i.e., NO), Sweden (i.e., SE), and Switzerland (i.e., CH) are nearly 90%. This indicates that the selected feature variables in the OLR model fit these countries better than the baseline model of the native group. The performances of the models in countries, such as France (i.e., FR), Hungary (i.e., HU), Poland (i.e., PL), and Portugal (i.e., PT), are lower than the average. This indicates that the selected feature variables are not ideal.

**Table 4:** The performance metrics of OLR models.

	**Native groups**						**Immigrant groups**					
	**ACC_train**	**ACC_test**	**ACC_avg**	**Macro-F1**	**Macro-Precision**	**Macro-Recall**	**ACC_train**	**ACC_test**	**ACC_avg**	**Macro-F1**	**Macro-Precision**	**Macro-Recall**
Total	0.8271	0.8271	0.8271	0.5666	0.6573	0.5269	0.8112	0.8197	0.8155	0.5391	0.6370	0.5006
BE	0.8862	0.8821	0.8842	0.5506	0.6241	0.5119	0.8203	0.7957	0.8080	0.5506	0.5747	0.4330
FI	0.9193	0.9171	0.9182	0.5867	0.6714	0.5402	0.8757	0.8645	0.8701	0.5500	0.5849	0.5299
FR	0.7675	0.7812	0.7743	0.5146	0.6238	0.4911	0.7518	0.7542	0.7530	0.5183	0.5752	0.4914
DE	0.8177	0.8172	0.8175	0.6122	0.6816	0.5750	0.8126	0.7906	0.8016	0.5843	0.6424	0.5547
HU	0.6637	0.6654	0.6645	0.6108	0.6498	0.5931	0.7252	0.6545	0.6899	0.3833	0.3711	0.3981
IE	0.8142	0.8192	0.8167	0.5931	0.6629	0.5566	0.8223	0.8050	0.8136	0.5606	0.5988	0.5396
NL	0.9124	0.8987	0.9055	0.5310	0.6119	0.4923	0.8482	0.8367	0.8425	0.4620	0.4977	0.4448
NO	0.8979	0.8938	0.8958	0.5584	0.7130	0.5038	0.8813	0.8711	0.8762	0.5947	0.6859	0.5468
PL	0.7614	0.7666	0.7640	0.6199	0.6827	0.5880	0.7841	0.8108	0.7975	0.5229	0.5282	0.5188
PT	0.6920	0.6754	0.6837	0.5334	0.6526	0.5114	0.6912	0.7389	0.7151	0.4567	0.4470	0.4669
SI	0.7965	0.7982	0.7974	0.6145	0.6889	0.5812	0.8011	0.7699	0.7855	0.5722	0.6276	0.5468
ES	0.8228	0.8276	0.8252	0.5439	0.6471	0.5046	0.7914	0.8123	0.8019	0.5529	0.6346	0.5144
SE	0.8968	0.8883	0.8925	0.5922	0.6855	0.5439	0.8687	0.8511	0.8599	0.6341	0.7802	0.5703
CH	0.9065	0.9155	0.9110	0.5382	0.6161	0.4973	0.8632	0.8724	0.8678	0.5527	0.7364	0.4967
GB	0.8335	0.8319	0.8327	0.6187	0.6883	0.5794	0.8104	0.8445	0.8275	0.5775	0.6393	0.5470
Average	0.8260	0.8253	0.8256	0.5741	0.6598	0.5373	0.8099	0.8057	0.8078	0.5383	0.5976	0.5062

Notes: ACC_train = accuracy of training dataset; ACC_test = accuracy of test dataset; ACC_avg = average accuracy; The values of Macro-F1, Macro-Precision, and Macro-Recall are calculated on the basis of test dataset.

A geographical feature is that the countries with good performance of models are located in Western Europe (Belgium, Netherlands, Switzerland) and Northern Europe (Finland, Norway, Sweden). Hungary and Poland are two Eastern European countries with poor performance models. This phenomenon indicates that determinants of happiness for individual countries may need to be reconsidered with specific conditions of countries, such as the geographic location and the economic development.

In Table 4, the average performance of native groups’ models is better than that of immigrant groups’ models. Only the models of Finland and Norway maintain a high accuracy of nearly 90%. The model of the immigrant group in Belgium has a declining performance with a 6.69% accuracy loss.

From the results of OLR models in the native group, immigrant group, and subgroup of individual countries, it can be concluded that the selected feature variables should be further considered. The reason is that the determinants of happiness may be inconsistent among the native group, immigrant group, and subgroup of individual countries.

### Artificial neural network

The ANN model is introduced to make a comparison with the OLR model. Before training the ANN model, it is recommended to normalize all variables (i.e., make the minimum value to 0 and the maximum value to 1). Since the weight parameters of the neural network will be affected by the values of a wide range of variables, it is not conducive to the training and prediction of the neural network. This paper uses the most common structure of the neural network with a single hidden layer.

The nnet package is selected to construct the neural network. This is because it can accept response variables as factor variables that have been set in the OLR model. Furthermore, it is of great significance to select the appropriate number of nodes in the hidden layer based on the background knowledge and experiments. A for-loop is created to run each model in the neuron number from 1 to 14. The optimal number of neurons can minimize the test error of the model. The number of iterations (i.e., maxit in the nnet package) is set to 2,000, which is a large enough number that ensures all models converge in the for-loop.

**Figure 3:**
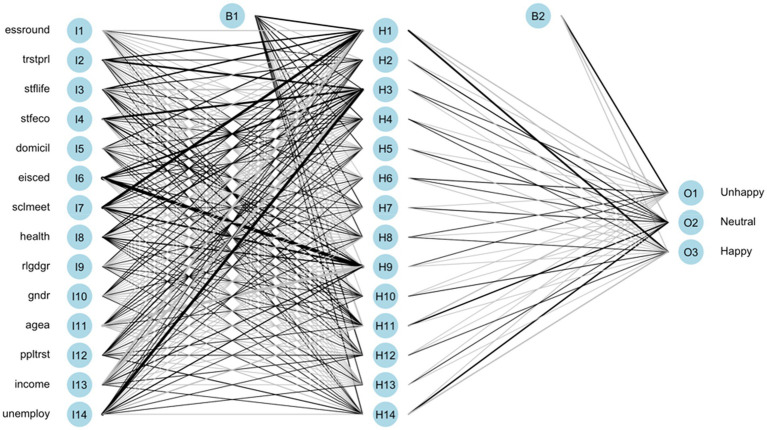
Trained ANN of the total native group.

**Figure 4:**
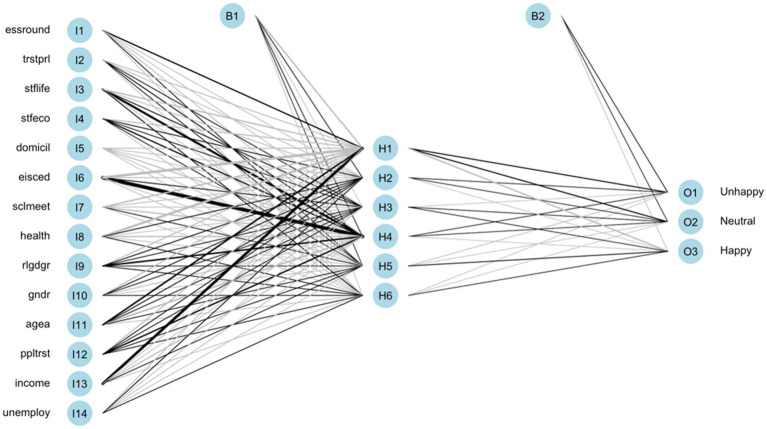
Trained ANN of the total immigrant group.

The ANN model is trained based on the above configurations. The illustration of the models with total native and immigrant samples are shown in Figures 3, 4, respectively. The black lines represent positive weights and the gray lines represent negative weights. The strength of weights is represented by the thickness of the line. The B1 and B2 are bias layers that apply constant values to the nodes, similar to the intercept terms of the regression model. Although the learning pattern of ANN can be visualized, the study of its structure cannot give usable conclusions about the function approximation. In other words, the relationship between the response variable and feature variables cannot be directly defined.

Although neural network models are described as “black boxes,” a sensitivity analysis can be used to rank the variable importance of the ANN model. Among a few methods that are capable of evaluating sensitivity in neural networks, the NeuralNetTools package is employed to compute the variable importance, which is based on the Lek profile method (Beck, 2018) that uses combinations of the absolute values of the weights. As shown in Table 5, the importance ranking order is different in each model of native groups. Notwithstanding, the top four important variables are similar among models. *Stflife*, *eisced,* and health are three of the most crucial factors that occupy the importance ranking of the native groups’ ANN models. Among these three factors, *stflife* is the most influential to natives’ happiness given that it appears in the importance rankings of all native groups. In addition, *health* can only stay in the rankings for half of the native groups, while *eisced* only fails to enter the rankings for native groups in GB and SE. The sensitivity analysis from the ANN model sheds more light on this matter that cannot be found in OLR models.

**Table 5:** Variable importance from native groups’ ANN models.

	Native groups			Immigrant groups		
	1st	2nd	3rd	1st	2nd	3rd
Total	stflife	eisced	agea	stflife	eisced	agea
BE	stflife	eisced	ppltrst	stflife	health	ppltrst
FI	eisced	stflife	agea	stflife	essround	domicil
FR	stflife	income	eisced	eisced	stflife	trstprl
DE	stflife	eisced	agea	stflife	eisced	agea
HU	eisced	stflife	income	stflife	rlgdgr	gndr
IE	stflife	health	eisced	stflife	health	sclmeet
NL	stflife	health	eisced	stflife	health	income
NO	stflife	health	eisced	stflife	health	income
PL	stflife	eisced	health	stflife	sclmeet	unemploy
PT	stflife	eisced	stfeco	eisced	stflife	agea
SI	stflife	eisced	health	stflife	health	ppltrst
ES	stflife	eisced	trstprl	stflife	stfeco	ppltrst
SE	stflife	income	agea	stflife	ppltrst	sclmeet
CH	stflife	unemploy	eisced	stflife	stfeco	health
GB	stflife	income	ppltrst	stflife	eisced	stfeco

The situation of immigrant groups is different from that of native groups. In Table 5, the top four important happiness determinants of the immigrant groups are diverse. Although *stflife*, *eisced,* and *health* are still three of the most important variables for immigrant groups, there are fewer models with the same factors in the importance ranking. Only *stflife* remains in the importance rankings among all immigrant groups. For immigrant groups, *health* appears more frequently in the importance ranking than *eisced*, which is contrary to the native groups.

Compared with the OLR model, the overall performance of the ANN model in the native group is better. The individual countries that have good performance with OLR are still outstanding and even better when using the ANN. However, the countries with the poor performance of the OLR models have been improved with the ANN. In particular, the average accuracy of ANN for Portugal has increased most by 4.2281%. In addition, the moderate macro F1 and recall of ANN are 2.8240% and 4.3635% greater than that of OLR. This indicates that the ANN performs better than the OLR in classifying each happiness level (Table 6).

Although the overall performance of the model for native groups has improved, the model for immigrant groups of individual countries, such as FI, HU and PL, is overfitted. Neural networks with a small sample size can easily memorize the data and cause overfitting. Therefore, large sample size is needed in most cases to train the ANN model. The immigrant sample sizes of FI, HU and PL are the smallest among all countries, with 517, 186, and 125, respectively. The accuracy gaps between their training and test datasets are the largest, 18.9538%, 23.4341%, and 30.4295%, respectively. The accuracy gaps between their training and test datasets increase as the sample size decreases (Table 6).

**Table 6:** The performance metrics of ANN models.

	**Native groups**							**Immigrant groups**						
	**Nodes**	**ACC_** **train**	**ACC_** **test**	**ACC_** **avg**	**Macro-** **F1**	**Macro-Precision**	**Macro-** **Recall**	**Nodes**	**ACC** **_train**	**ACC** **_test**	**ACC** **_avg**	**Macro-** **F1**	**Macro-** **Precision**	**Macro-Recall**
Total	14	0.8362	0.8340	0.8351	0.6243	0.6593	0.6011	6	0.8281	0.8188	0.8234	0.5773	0.6211	0.5510
BE	2	0.8859	0.8794	0.8827	0.4599	0.4684	0.4542	2	0.8388	0.8026	0.8207	0.5191	0.5173	0.5436
FI	9	0.9285	0.9215	0.9250	0.6379	0.6606	0.6224	8	0.9972	0.8077	0.9025	0.5597	0.5755	0.5477
FR	4	0.7838	0.7685	0.7762	0.5592	0.6410	0.5317	2	0.7771	0.7048	0.7409	0.5079	0.6154	0.4877
DE	7	0.8244	0.8219	0.8232	0.6176	0.6456	0.5966	2	0.8310	0.8173	0.8242	0.5709	0.5856	0.5595
HU	9	0.6892	0.6628	0.6760	0.6131	0.6231	0.6060	4	0.9308	0.6964	0.8136	0.4198	0.4225	0.4228
IE	3	0.8200	0.8266	0.8233	0.6484	0.6710	0.6303	1	0.8148	0.8130	0.8139	0.6429	0.7100	0.6032
NL	2	0.9080	0.9203	0.9141	0.6596	0.6844	0.6386	6	0.9081	0.7943	0.8512	0.4215	0.4702	0.4101
NO	4	0.9035	0.8946	0.8990	0.5846	0.6834	0.5461	1	0.9044	0.8829	0.8936	0.5278	0.7962	0.5126
PL	2	0.7696	0.7661	0.7678	0.6538	0.6533	0.6546	8	0.9885	0.6842	0.8364	0.4703	0.4660	0.4762
PT	11	0.7428	0.7091	0.7260	0.6053	0.6365	0.5870	2	0.7357	0.6575	0.6966	0.4801	0.4808	0.4877
SI	1	0.8017	0.8025	0.8021	0.6151	0.6745	0.5930	1	0.7756	0.8000	0.7878	0.5121	0.4997	0.5253
ES	3	0.8323	0.8184	0.8253	0.5783	0.6447	0.5447	6	0.8921	0.7742	0.8332	0.5676	0.6273	0.5363
SE	3	0.8919	0.9051	0.8985	0.6466	0.7378	0.5939	12	0.9571	0.8404	0.8987	0.5222	0.5490	0.5182
CH	6	0.9136	0.9141	0.9138	0.5013	0.5959	0.4918	2	0.8838	0.8594	0.8716	0.6073	0.7440	0.5533
GB	3	0.8345	0.8400	0.8373	0.6319	0.6854	0.6029	1	0.8291	0.7862	0.8077	0.5430	0.6167	0.5111
Average	-	0.8354	0.8303	0.8328	0.6023	0.6478	0.5809	-	0.8683	0.7837	0.8260	0.5281	0.5811	0.5154

Notes: Nodes are the optimal number of neurons in the single hidden layer; ACC_train = accuracy of training dataset; ACC_test = accuracy of test dataset; ACC_avg = average accuracy; The values of Macro-F1, Macro-Precision, and Macro-Recall are calculated on the basis of test dataset.

## Discussion

People have migrated in pursuit of happiness for thousands of years. Migration to another country has a significant psychological impact on the individual, however, it is not solely a personal issue because it also has an influence on the sociocultural, civic-political and economic development of both the origin and destination countries. EU countries host a large number of international migrants, and their policymakers are becoming increasingly aware that the happiness level of immigrants can have a ripple effect on individuals, households, communities and, ultimately, countries.

The purpose of the current study is twofold: (i) to study the differences in happiness factors between immigrants and natives through single-country and cross-country modeling; (ii) to evaluate the performance of machine learning techniques on predicting the happiness levels of immigrant groups and native groups. To this end, we employ a total sample size of 196,724 respondents to establish the OLR model and the ANN model *via* using the ESS data of 15 countries in nine survey rounds.

The results show that the determinants of happiness are different among the immigrant groups, native groups, and even in each country surveyed. Consistent with previous studies (MacKerron, 2012; Hendriks, 2015; Steptoe et al., 2015), the sensitivity analysis shows that satisfaction with life, subjective general health, and the highest level of education are the three most prominent determinants that contribute to one’s happiness. With the advantages of the ANN method, we further find that subjective general health is more important than education for immigrants, and education is more important than subjective general health for natives. Therefore, it is necessary to build different models for immigrants and natives in each country.

As regards the performance of prediction models, the overall accuracies of OLR and ANN baseline models in both immigrant groups and native groups are >80%. A lower error can be achieved through a case-by-case analysis. In addition, the evaluation results of each model indicate that the prediction performance of ANN is better than OLR. This is congruent with the results of Weber et al. (2018). However, ANN may lead to overfitting for a small sample of immigrant groups, which partially supports prior research (Dreiseitl and Ohno-Machado, 2002) stating that artificial neural networks are subject to the pervasive trade-offs between flexibility and overfitting.

Some theoretical and practical implications should be noted. First, this paper has demonstrated that the research of migration and happiness can benefit from the knowledge acquired using cutting-edge machine learning methods with social survey data. Although a number of quantitative research have been performed to advance the understanding of the happiness-gap between immigrants and natives (Hendriks, 2015), this is the first time that machine learning algorithms are applied to the study of this field. The application of machine learning methods, especially artificial neural network, can provide quantitative insights into the relative importance of happiness factors. Therefore, researchers and policymakers need to understand that the machine learning approach is capable of revealing previously unknown relationships and thereby allows us to better understand the decision-making processes and construct better policies. Second, the study of happiness has remained exceptionally data-driven, while prior studies tend to use self-collected samples (Li et al., 2016; Pérez-Benito et al., 2019; Islam and Goldwasser, 2020), resulting in a dispersed field in which few scholars build on each other’s work. One of our insights is that the combination of the machine learning approach with a huge amount of microdata coming from the same dataset enables scholars to replicate results and make attempts to boost prediction accuracy based on others’ research.

While the findings reported here represent a contribution to the literature, the present study bears certain limitations. Therefore, further theoretical and empirical work is needed to improve the performance of machine learning methods in the research of migration and happiness.

First, due to the lack of immigrant samples in particular countries, such as Hungary and Poland, the machine learning algorithms may be overfitted. Future research should concentrate on the sampling of immigrants to avoid overfitting. Second, the factor gender is included as one of the determinants in our study that has not been examined as a mutual determinant in both native and immigrant groups across countries. The gender difference is an intriguing point worth further investigation, but due to the limitations of ESS data, whose samples are newly selected in each round and do not provide time-varying information like longitudinal data, we were unable to investigate it in depth. It could be a promising venue for future research to employ longitudinal data from a specific country (e.g., the UK’s Understanding Society data) that would allow us to examine the gender difference and the generations of immigrants. Third, factors like cultural distance, cultural diversity, and discrimination can be taken into account to improve the happiness level prediction model for immigrants. Bobowik et al. (2022) examined the linkage of global identification and ethnocultural diversity with the social network data of immigrants, where they found that the ethnocultural diversity among strong contacts was associated with global identification. Though there is a negative relationship between discrimination and SWB in immigrant groups, the relationship weakens when comparison strategies are considered, implying that how immigrants cope with discrimination under comparison processes may affect their SWB accordingly (Madi et al., 2022).

## Data availability statement

Publicly available datasets were analyzed in this study. This data can be found at: https://doi.org/10.21338/NSD-ESS-CUMULATIVE.

## Author contributions

YL, SC, and MY: conceptualization and writing—review and editing. YL and SC: methodology and formal analysis. YL: software, data curation, and writing—original draft preparation. SC: supervision and funding acquisition. All authors contributed to the article and approved the submitted version.

## Funding

This research was supported by Foundation for Distinguished Young Talents in Higher Education of Guangdong, China (Grant No. 2019WQNCX15).

## Conflict of interest

The authors declare that the research was conducted in the absence of any commercial or financial relationships that could be construed as a potential conflict of interest.

## Publisher’s note

All claims expressed in this article are solely those of the authors and do not necessarily represent those of their affiliated organizations, or those of the publisher, the editors and the reviewers. Any product that may be evaluated in this article, or claim that may be made by its manufacturer, is not guaranteed or endorsed by the publisher.

## Figures and Tables

**Table A1:** Full names of investigated countries

Country code	Full names
BE	Belgium
FI	Finland
FR	France
DE	Germany
HU	Hungary
IE	Ireland
NL	Netherlands
NO	Norway
PL	Poland
PT	Portugal
SI	Slovenia
ES	Spain
SE	Sweden
CH	Switzerland
GB	United Kingdom

## Footnotes

^1^ https://www.consilium.europa.eu/en/press/press-releases/2019/10/24/economy-of-wellbeing-the-council-adopts-conclusions/
